# Effects of nitrogen deposition on the rhizosphere nitrogen-fixing bacterial community structure and assembly mechanisms in *Camellia oleifera* plantations

**DOI:** 10.3389/fmicb.2024.1414724

**Published:** 2024-06-18

**Authors:** Caixia Liu, Zhilong He, Yongzhong Chen, Yanming Xu, Wei Tang, Longsheng Chen

**Affiliations:** ^1^Research Institute of Oil Tea Camellia, Hunan Academy of Forestry, Changsha, China; ^2^National Engineering Research Center for Oil Tea Camellia, Changsha, China

**Keywords:** nitrogen deposition, *Camellia oleifera*, nitrogen-fixing bacteria, community structure, community assembly

## Abstract

Increased nitrogen deposition is a key feature of global climate change, however, its effects on the structure and assembling mechanisms of the nitrogen-fixing bacteria present at the root surface remain to be elucidated. In this pursuit, we used NH_4_NO_3_ to simulate nitrogen deposition in a 10-year-old *Camellia oleifera* plantation, and set up four deposition treatments, including control N0 (0 kg N hm^−2^ a^−1^), low nitrogen N20 (20 kg N hm^−2^ a^−1^), medium nitrogen N40 (40 kg N hm^−2^ a^−1^) and high nitrogen N160 (160 kg N hm^−2^ a^−1^). The results showed that nitrogen deposition affected the soil nitrogen content and the structure of the nitrogen-fixing bacterial community. Low nitrogen deposition was conducive for nitrogen fixation in mature *C. oleifera* plantation. With increasing nitrogen deposition, the dominant soil nitrogen-fixing bacterial community shifted from Desulfobulbaceae to Bradyrhizobium. When nitrogen deposition was below 160 kg N hm^−2^ a^−1^, the soil organic matter content, total nitrogen content, nitrate nitrogen content, ammonium nitrogen content, urease activity, soil pH and nitrate reductase activity influenced the composition of the nitrogen-fixing bacterial community, but the stochastic process remained the dominant factor. The results indicate that the strains of *Bradyrhizobium japonicum* and *Bradyrhizobium* sp. ORS 285 can be used as indicator species for excessive nitrogen deposition.

## 1 Introduction

Nitrogen deposition is an important pathway for nitrogen input in the biosphere (Guo et al., 2024). Atmospheric nitrogen deposition has increased rapidly in recent years due to the combustion of fossil fuel, application of chemical fertilizers, livestock development, etc. Ackerman estimated the interannual changes in the global dry and wet nitrogen deposition between 1984 and 2016, and found that most regions of the world showed an upward trend in nitrogen deposition (Ackerman et al., 2019; Song et al., 2021; Shang et al., 2022). China has the highest nitrogen deposition in the world, and high nitrogen deposition is mainly distributed in the east, centre and south. The mean nitrogen deposition in the south is >30 kg N hm^−2^ a^−1^, with the highest value exceeding 60 kg N hm^−2^ a^−1^ (Liu M. et al., 2019; Xie et al., 2023).

Nitrogen-fixing flora is an important functional flora in forest ecosystems that lives in the forest soil. They reduce the atmospheric nitrogen to ammonia, provide effective nitrogen for plant growth, maintain soil fertility and increase the plant productivity in forest ecosystems (Zhao et al., 2020). However, there is no consistent conclusion on the effect of nitrogen deposition on the community structure and diversity of nitrogen-fixing bacteria. Orr’s research showed that nitrogen addition increases the soil organic carbon content and the abundance of nitrogen fixing bacteria, thereby promoting the nitrogen fixation function of soil microorganisms (Orr et al., 2012). Numerous research results have shown that nitrogen deposition reduces the plant and microbial diversity (Dai et al., 2018; Midolo et al., 2019), leading to changes in the structure of nitrogen-fixing microbial communities, which alters the soil nitrogen cycle and the rate of nitrogen fixation, and ultimately affects the plant growth and soil health (Jianhong et al., 2017). Zheng studied the response of soil nitrogen fixation to nitrogen addition in different types of subtropical forests in Dinghushan, China. The results showed that nitrogen addition significantly increased the NH_4_^+^ content in the disturbed forest soil, thereby inhibiting the nitrogenase activity. However, the response of nitrogen fixing microbial communities was not clearly elucidated (Zheng et al., 2017). Nitrogen enrichment in soil affects the relative importance of deterministic and stochastic processes in the nitrogen-fixing bacterial community assembly, thereby influencing the trajectory of community aggregation (Liu S. et al., 2021; Liu W. et al., 2021). Disentangling the underlying mechanisms of nitrogen-fixing bacterial community establishment and maintenance is critical to decipher the microbial community response and feedback to changes in the environment.

*Camellia oleifera* is an important woody oil-bearing tree species in China, with a current national planting area of 4.53 million hectares and a tea oil output of 627,000 tons, bringing the total industrial value to 116 billion yuan (Chen et al., 2022). Hunan, as a major *C. oleifera* producing area, also maintains a high level of nitrogen deposition, making it of great significance to maintain high and stable production under nitrogen deposition conditions. This study explores the impact of nitrogen deposition on the structure and function of nitrogen-fixing bacterial communities. Specifically, the response strategies of nitrogen-fixing bacterial communities to nitrogen feedback regulation were deciphered in conditions with increased nitrogen content of both the air and soil. Our results can provide a reference in the management of *C. oleifera* plantations under environmental changes.

## 2 Materials and methods

### 2.1 Overview of the study area

The experimental site was located in Lukou Town, Changsha City, Hunan Province (latitude 28°22′, longitude 113°11′). The area was characterized by a subtropical monsoon humid climate, with an average annual temperature of 16.8 to 17.2°C, average frost-free period of 275 days, and 1361.6 mm average annual precipitation. The soil was lateritic, formed from argillaceous slate and quaternary red earth, with a soil pH of 4.65 and organic matter content of 11.85 g·kg ^−1^.

### 2.2 Experimental design

Twelve 10 m × 10 m plots of 10-year-old *C. oleifera* plantations with consistent growth conditions were set up to simulate the nitrogen deposition, with a 10 m wide buffer strip between the plots to prevent mutual influence. Based on the actual nitrogen deposition of 30 kg N hm^−2^ a^−1^ in the subtropical region of China, four levels of nitrogen deposition treatments were set up, namely, control (N0, 0 kg N hm^−2^ a^−1^), low nitrogen (N20, 20 kg N hm^−2^ a^−1^), medium nitrogen (N40, 40 kg N hm^−2^ a^−1^), and high nitrogen (N160, 160 kg N hm^−2^ a^−1^) (Cui et al., 2014; Du et al., 2017). Three replicate sample plots were set up for each N application level. Simulated N deposition spraying was started from January 2020, and the total experimental period was of 2 years. The annual amount of N applied to each sample plot was equally divided into 6 equal parts and sprayed every other month, while the control sample plots were sprayed with water.

### 2.3 Experimental methods

In May 2022, in each plot, six *C. oleifera* trees were selected in an S-pattern. Soil samples were collected from the 0–20 cm soil layer using a soil auger, approximately 50 cm from the base of the *C. oleifera* trunks. The samples from six points were mixed together and stored in a portable freezer at −20°C. The soil samples were sent to the laboratory, and the plant tissues, roots, and rocks were sieved out using a 2 mm sieve. Samples for DNA extraction were frozen and stored at −80°C, while the remaining soil was air-dried in a cool place for further chemical analysis.

The soil organic matter content (SOM) was determined by the potassium dichromate oxidation spectrophotometric method (van Gaans et al., 1995); total nitrogen content was measured by the Kjeldahl method (Tsiknia et al., 2014); soil ammonium nitrogen was extracted with potassium chloride and determined by the distillation-colorimetric method; soil nitrate nitrogen was determined by the phenol disulfonic acid colorimetric method (Hale et al., 2013); and the soil nitrogen transformation-related enzyme activities (catalase, urease, nitrate reductase, and nitrogenase activities) were determined using spectrophotometry and enzyme activity test kits provided by Beijing Solarbio Science & Technology Co., Ltd. Total soil DNA was isolated and purified using the Powersoil DNA Isolation Kit (MoBio, Carlsbad, CA, United States). The diversity of nitrogen-fixing bacteria was determined by amplifying the nifH gene using primers nifH-F 5’ AAAGGYGGWATCGGYAARTCCACCAC 3′ and nifH-R 5’ TTGTTSGCSGCRTACATSGCCATCAT 3′, with PCR amplification parameters: 94°C for 5 min; [94°C for 30s, 58°C for 30s, 72°C for 60s] for 35 cycles; 72°C for 7 min. PCR products were checked for target band size using 1% agarose gel electrophoresis and purified with the Agencourt AMPure XP nucleic acid purification kit. The PCR products were used to construct a microbial diversity sequencing library and sequenced on the Illumina MiSeq PE300 high-throughput sequencing platform at Beijing Novogene Technology Co., Ltd. for paired-end sequencing.

### 2.4 Data analysis

The sequencing results were quality filtered and assembled into high-quality sequences using Flash and Trimmomatic software, resulting in valid data. The OTU representative sequences were aligned and analyzed using the RDP Classifier algorithm, and the species information of their communities was annotated at various levels. The Alpha diversity indices and Beta diversity distance matrices of the samples were calculated using the QIIME software. The differences in soil nitrogen content, enzyme activities, and nitrogen-fixing bacterial diversity among the different treatments were assessed using the Tukey HSD multiple comparison test (Li et al., 2018). The zero-model method was used to calculate the β-nearest taxon index (βMNTD), so as to infer the community aggregation processes (Xun et al., 2021). Indicator species and network analyses were used to study the bacterial groups sensitive to nitrogen deposition (Jiao et al., 2020). Further, we used the functions included in the package indicspecies to conduct the indicator species analysis, and thodcombines the species relative abundance with the relative frequency of occurrence of the species in different habitats (Babko et al., 2022). The relationship between the nitrogen-fixing bacterial community and environmental factors was examined using the Mantel test (Chen et al., 2022). Data analysis was performed using R Core Team (v. 4.2.1).

## 3 Results and analyses

### 3.1 Effect of nitrogen deposition on soil organic matter and nitrogen content

**Figure 1:**
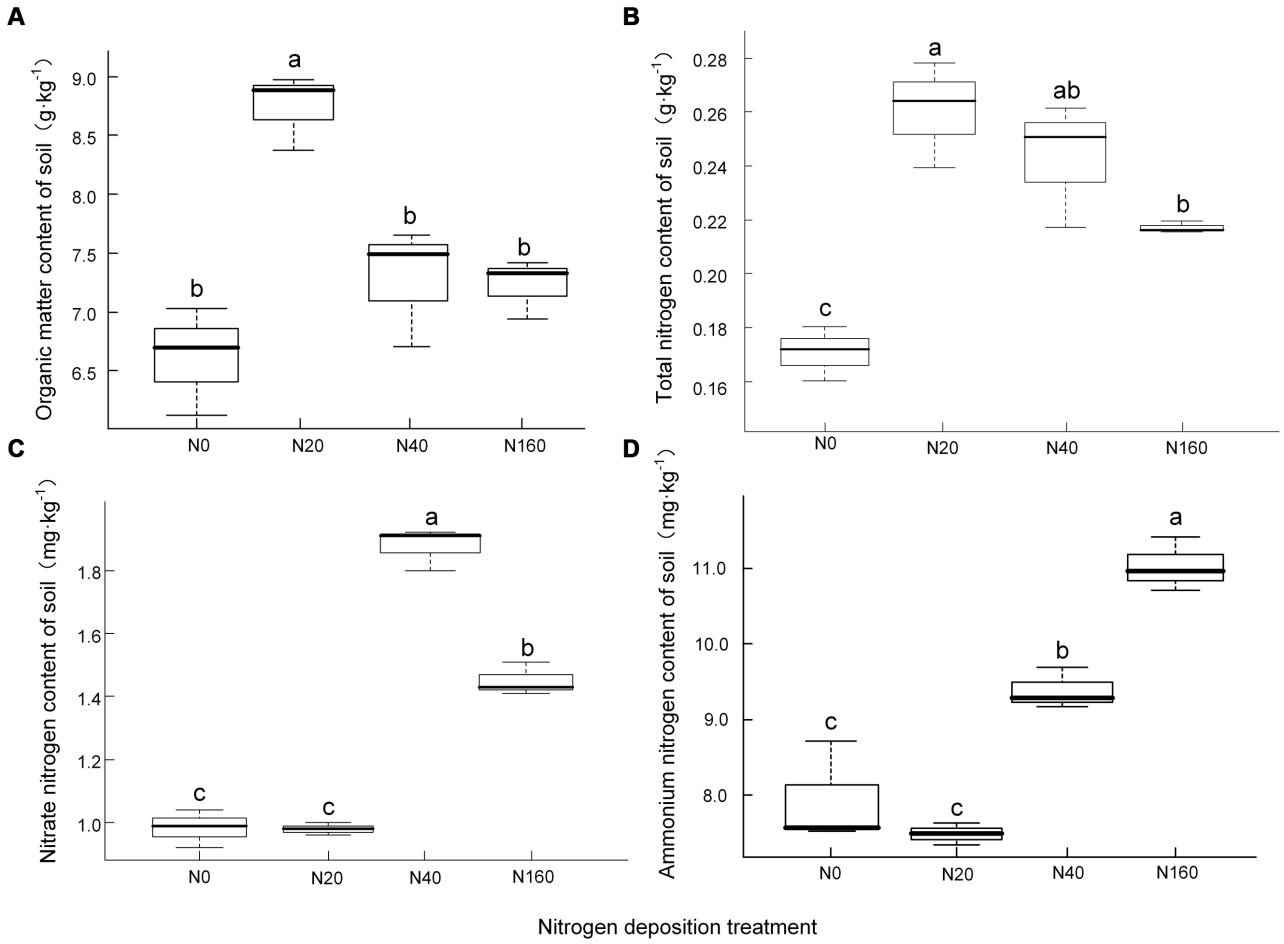
Soil organic matter **(A)** and nitrogen content **(B-D)** under nitrogen deposition treatments. **(A)** Organic matter content of soil, **(B)** Total nitrogen content of soil, **(C)** Nitrate nitrogen content of soil, **(D)** Ammonium nitrogen content of soil. Different lowercase letters indicate significant differences at *P*<0.05.

The content of organic matter and total nitrogen in the soil showed an initial increasing trend followed by a decrease with an increase in the nitrogen deposition concentration, reaching their maxima of 8.74 g·kg ^−1^ and 0.26 g·kg ^−1^, respectively, under the N20 treatment. There were no significant differences between the N40 and N160 treatments, indicating that when the amount of nitrogen deposition exceeded a certain value, further nitrogen deposition did not significantly increase the content of soil organic matter and total nitrogen, instead it showed a decreasing trend (Figure 1). The content of nitrate nitrogen in the soil exhibited an initial increasing trend followed by a subsequent decrease with an increase in the nitrogen deposition, and reached its maximum value of 1.88 mg·kg ^−1^ under the N40 treatment. The soil nitrate nitrogen content under the N160 treatment exhibited a clear decreasing trend. The content of ammonium nitrogen in the soil was significantly positively correlated with the amount of nitrogen deposition, reaching its maximum value at N160, with a value of 11.03 mg·kg ^−1^.

### 3.2 Effects of nitrogen deposition on soil enzyme activities in *Camellia oleifera*

**Figure 2:**
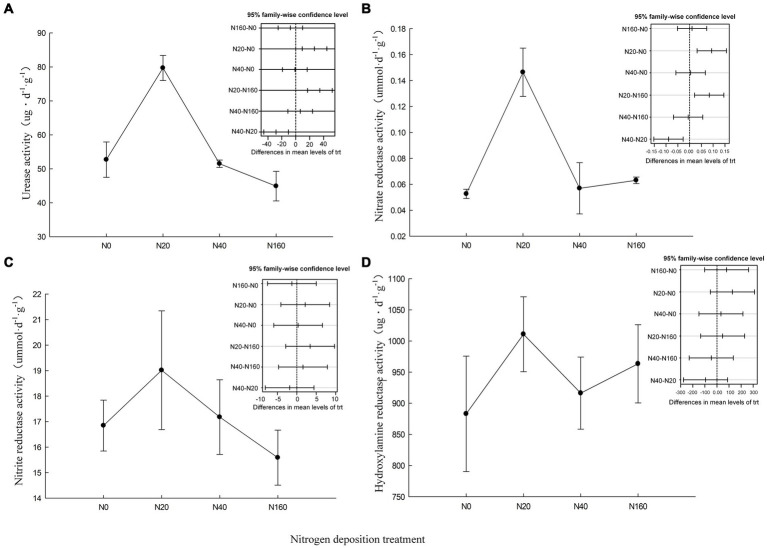
Nitrogen cycling related enzyme activities in soil under nitrogen deposition treatment. **(A)** Urease activity, **(B)** Nitrate reductase activity, **(C)** Nitrite reductase activity, **(D)** Hydroxylamine reductase activity.

The activity of the nitrogen transformation enzymes in soil is closely related to the productivity of *C. oleifera*. Urease hydrolyzes the carbon-nitrogen bonds in soil organic matter to produce ammonia, carbon dioxide, and water, thereby facilitating the conversion of organic nitrogen to available nitrogen. Among the nitrogen deposition treatments, only N20 significantly increased the activity of urease in the soil of *C. oleifera*, while the N160 treatment reduced the activity of urease in the soil, consistent with previous studies where excessive nitrogen addition the inhibited urease activity (Qian et al., 2012). Soil nitrification and denitrification are catalyzed by nitrate reductase and nitrite reductase, and the activity of nitrate reductase was significantly higher under the N20 treatment than other treatments, but there was no significant difference in the nitrite reductase activity among the treatments (Figure 2). Hydroxylamine reductase reduces the intermediate product hydroxylamine formed in soil nitrogen metabolism (dissimilatory nitrate reduction or ammonia oxidation) to ammonia. It affects the volatilization loss of nitrogen and the emission of greenhouse gases in the soil nitrogen metabolism process. However, in this study, different nitrogen deposition levels did not have a significant effect on the activity of hydroxylamine reductase.

### 3.3 Effects of nitrogen deposition on the structure of nitrogen-fixing bacterial communities

**Figure 3:**
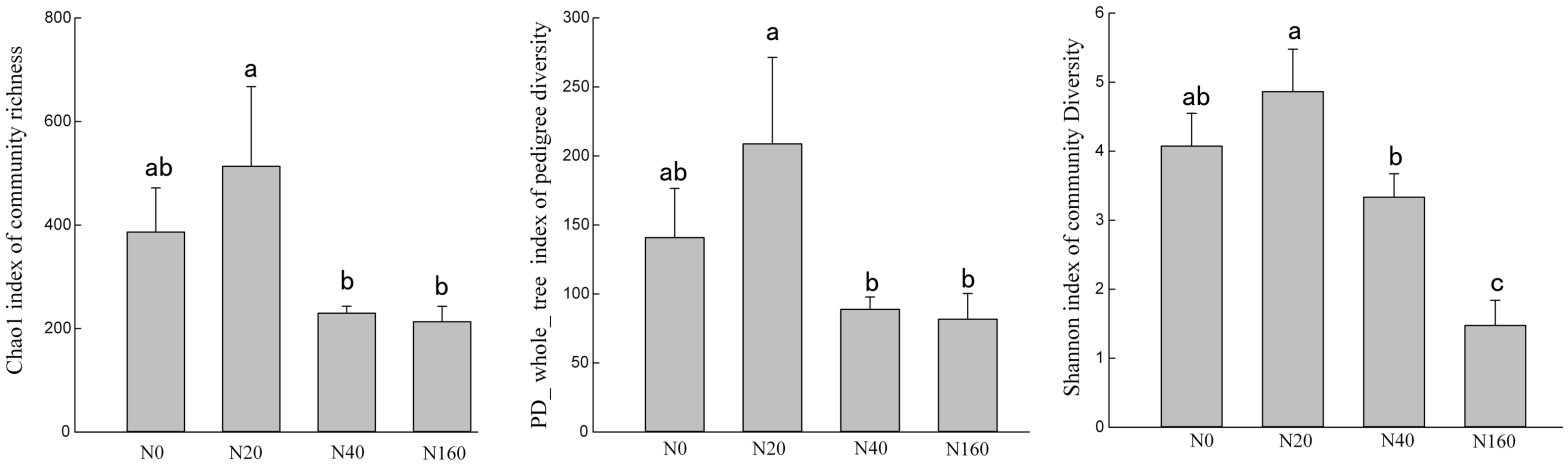
Nitrogen fixing bacteria α diversity of soil in nitrogen deposition treatment.

**Figure 4:**
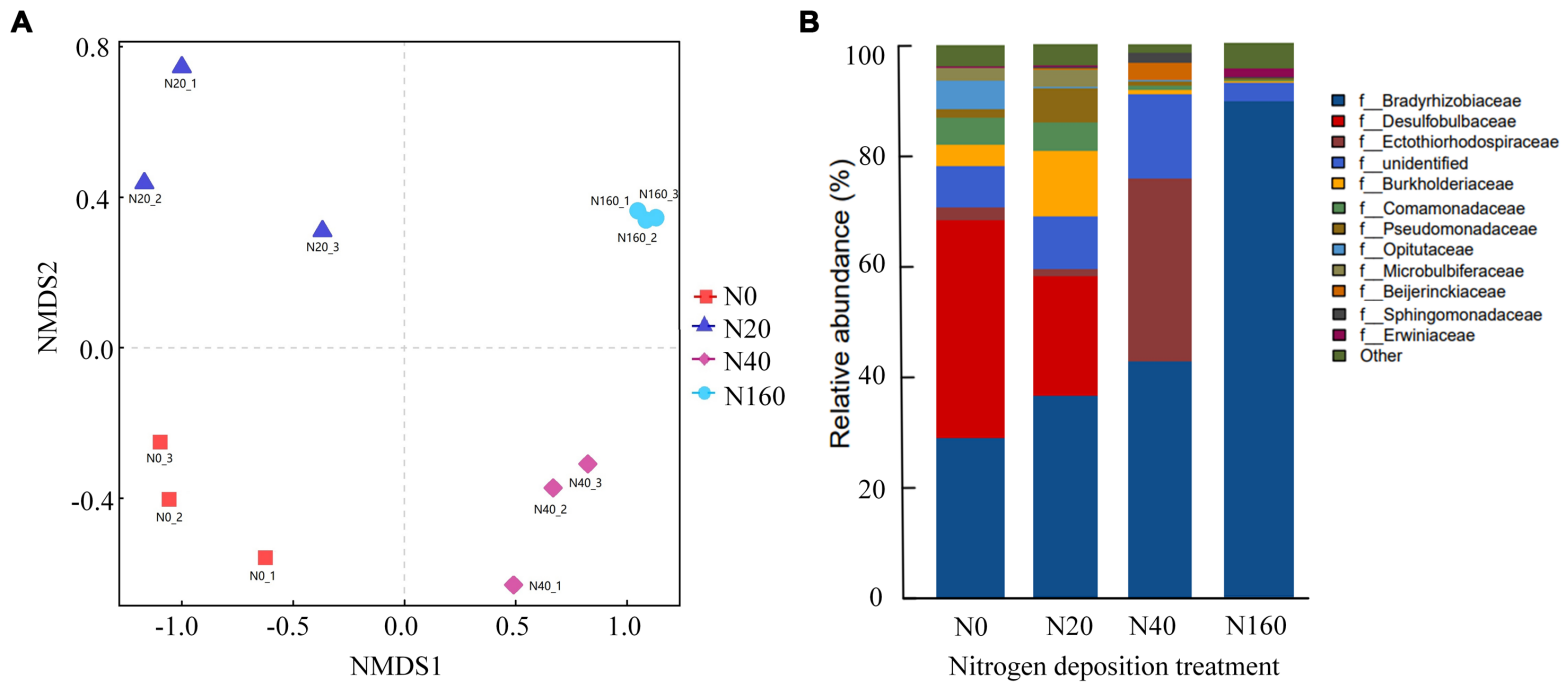
NMDS analysis of nitrogen fixing bacterial communities **(A)** and community composition at the family level **(B)** in nitrogen deposition treatment.

The results of Alpha diversity of nitrogen-fixing bacteria in different treatment soils indicated that the Chao1, PD-whole-tree, and Shannon indices followed generally a consistent trend across treatments. The richness, evenness, and phylogenetic diversity indices of the nitrogen-fixing bacteria followed the order of N20 > N0 > N40 > N160, indicating that low nitrogen deposition treatments enhanced the diversity of soil nitrogen-fixing bacteria, while medium and high nitrogen treatments reduced their diversity (Figure 3). Non-metric multidimensional scaling (NMDS) analysis of the nitrogen-fixing bacterial community structure in different soil samples showed that the four nitrogen treatments did not overlap, suggesting that the nitrogen deposition treatments affected the structure of soil nitrogen-fixing bacterial communities (Figure 4A). In all soil samples, nitrogen-fixing bacterial communities included 19 phyla, 32 classes, 69 orders, 112 families, and 210 genera. At the family level, the nitrogen-fixing bacteria with high abundance included Bradyrhizobiaceae, Desulfobulbaceae, Ectothiorhodospiraceae, Burkholderiaceae, etc. Among them, with the increase in the nitrogen deposition, the abundance of Bradyrhizobiaceae gradually increased, reaching 89.64% under the N160 treatment. However, the abundance of Desulfobulbaceae decreased with an increasing nitrogen deposition, from 39.55% in N0 treatment to 0.02% in N160 treatment. Notably, the abundance of Ectothiorhodospiraceae was significantly higher under the N40 treatment than other treatments, and the abundance of Erwiniaceae was significantly higher under the N160 treatment than other sites. In the N40 plots, 15.29% of the bacterial communities were not annotated, indicating the possible presence of unclassified new bacterial groups in the soil (Figure 4B).

### 3.4 Effects of nitrogen deposition on the assembly of nitrogen-fixing bacterial communities

**Figure 5:**
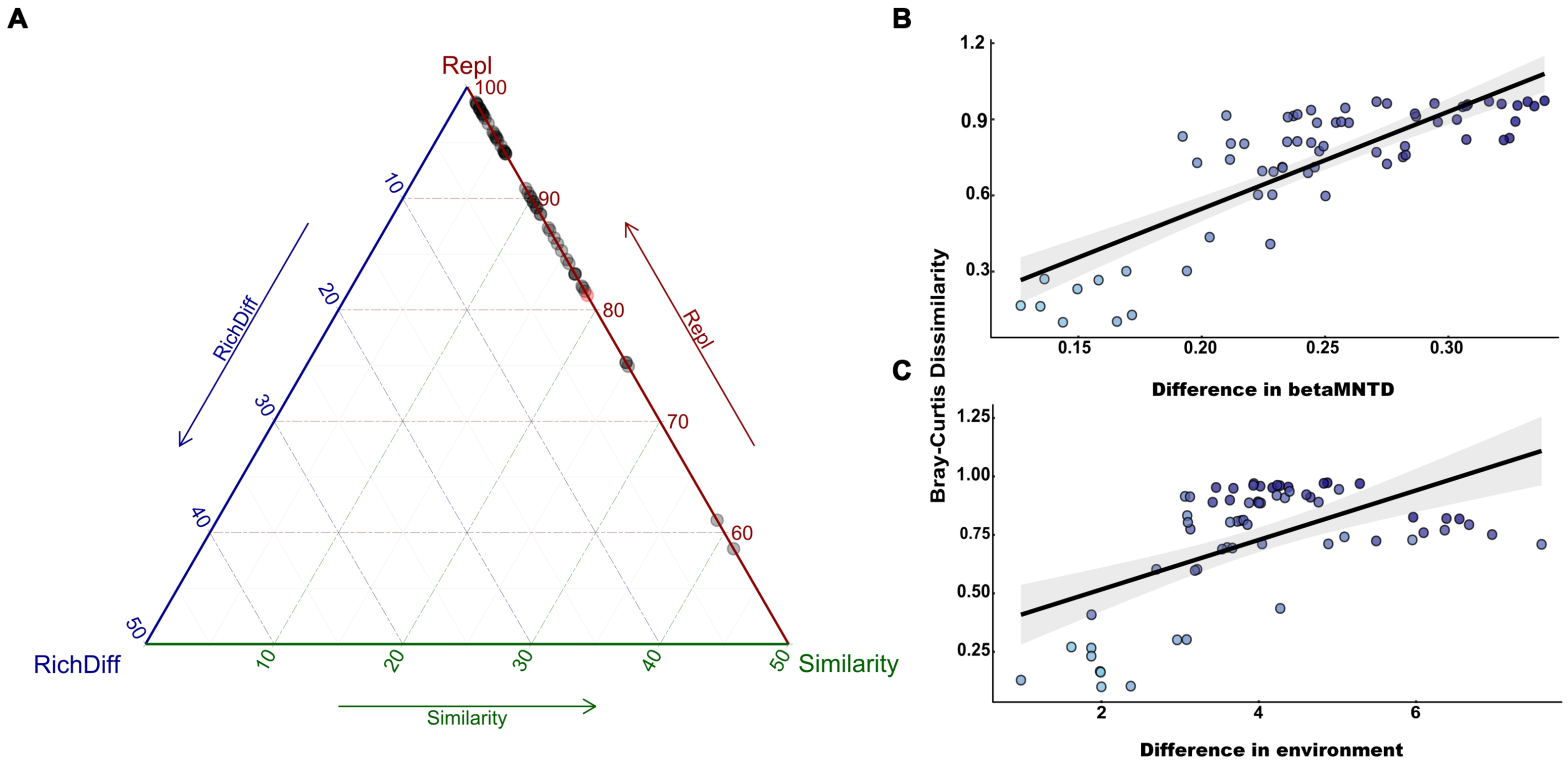
Nitrogen fixing bacteria β diversity analysis. **(A)** ternary plots of beta diversity components, **(B)** linear regression between β diversity and genetic distance, **(C)** linear regression between β diversity and environmental distance.

The beta diversity of nitrogen-fixing bacteria was further divided into total replacement diversity and total richness difference diversity. The results indicate that the differences in the composition of nitrogen-fixing bacterial communities were determined by the species replacement process, which accounted for 40.65% of the bacterial beta diversity (Figure 5A). Linear regression between beta diversity and environmental/genetic distance showed that the differences in the composition of soil nitrogen-fixing bacterial communities increased with an increase in the environmental/genetic distance (Figures 5B,C). The slope of the curve fitting bacterial diversity to genetic distance was higher than that to the environmental distance, indicating that stochastic processes dominated the assembly process of the bacterial communities. Furthermore, the Mantel tests and partial Mantel tests also indicated that beta diversity was significantly positively correlated with both the environmental and genetic distances, but the impact of genetic distance was more significant. This suggests that both deterministic and stochastic processes affected community establishment, but stochastic processes exhibited a greater impact.

### 3.5 Response of nitrogen-fixing bacterial communities in *Camellia oleifera* plantations to soil environment

**Figure 6:**
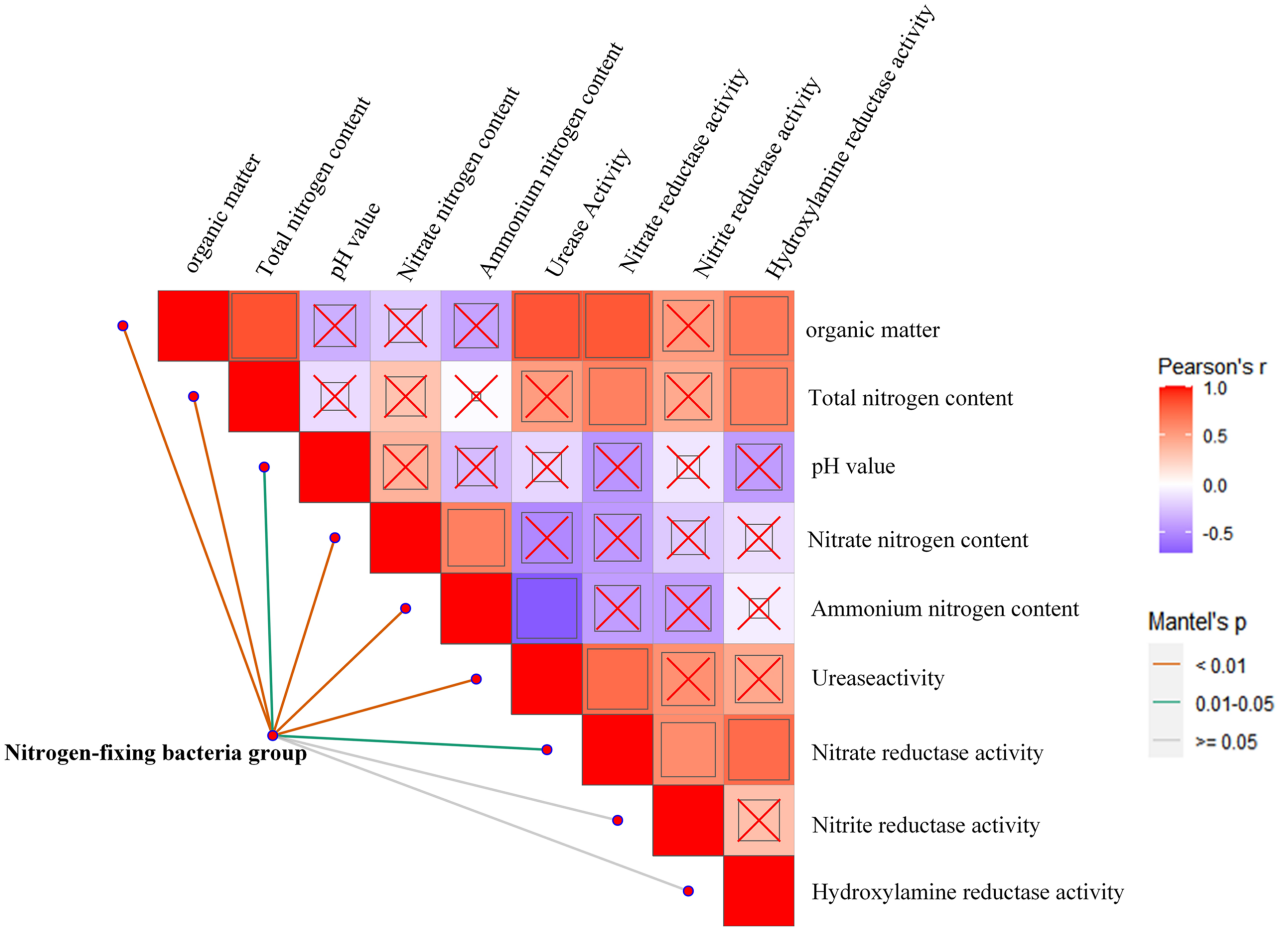
Environmental impact on nitrogen fixing bacteria communities. Pairwise comparisons of environmental factors are shown, with a color gradient denoting Spearman’s correlation coefficients, and ‘x’ indicates no significant correlation. Community composition of bins was related to each environmental factor by Mantel tests (betaMNTD-corrected). Edge width corresponds to the Mantel’s r statistic, and edge color denotes the statistical significance based on 9,999 permutations.

To further analyze the environmental driving factors of the composition of the nitrogen-fixing functional bacterial community, we correlated the differences in community composition (betaMNTD) with environmental factors. The results indicate that the soil environmental factor - pH was not significantly correlated with other soil environmental factors. The factor nitrate reductase activity was relatively active and changes significantly with variations in soil organic matter, total nitrogen content, urease activity, nitrite reductase activity, and hydroxylamine reductase activity (Figure 6). We also found that the soil organic matter content, total nitrogen content, nitrate nitrogen content, ammonium nitrogen content, and urease activity were highly significantly correlated with the structure of the nitrogen-fixing bacterial community, while soil pH and nitrate reductase activity was significantly related to the community structure of the nitrogen-fixing bacteria.

### 3.6 Core microbiome and indicator species of *Camellia oleifera* in response to nitrogen deposition

**Figure 7:**
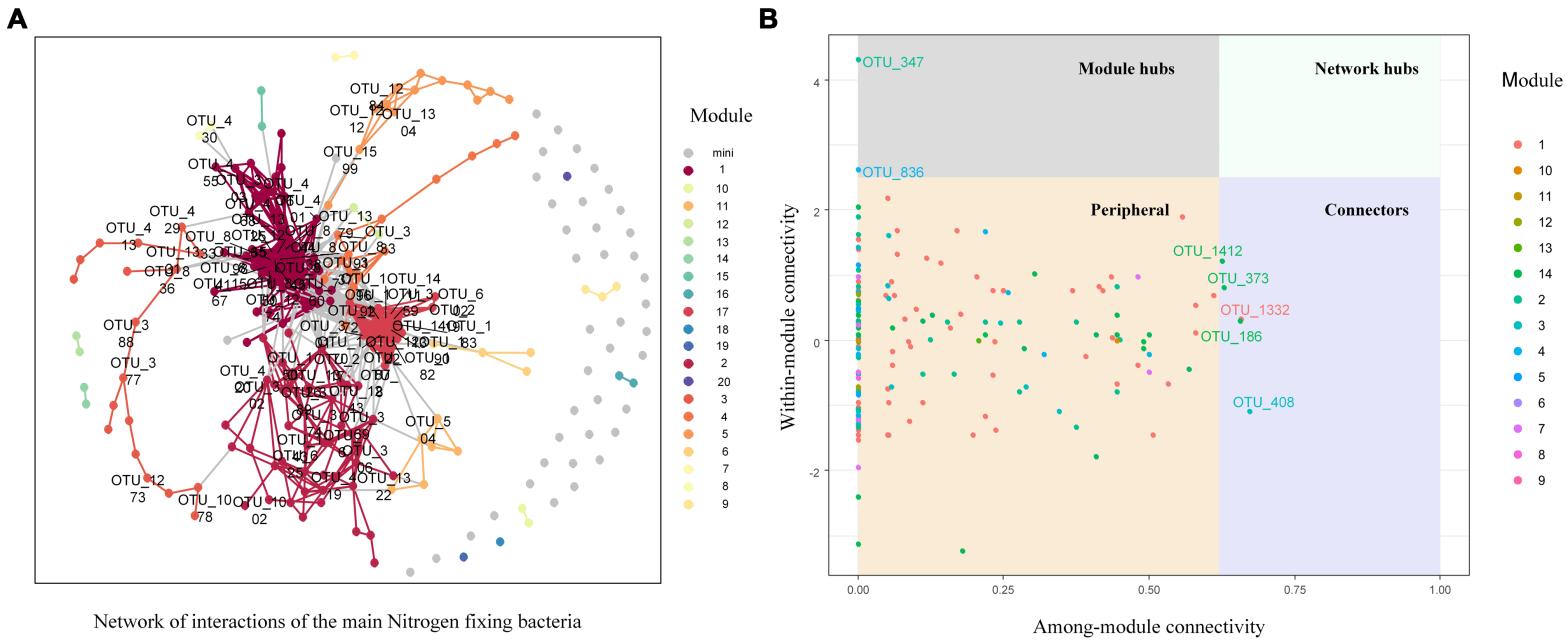
Nitrogen fixing bacteria network analysis of soil in nitrogen deposition treatment. **(A)** Network of co-occurring 90% cutoff OTUs based on correlation analysis. A connection stands for a strong (Spearman’s ρ>0.6) and significant (*P*-value <0.01) correlation. **(B)** network structure characteristics analysis.

The network of nitrogen-fixing bacterial communities in the soil of *C. oleifera* plantations was not complex, and comprised of 231 nodes and 1803 links, positive link ratio of 89.18%, average degree of connectivity of 15.61, average path length of 3.35, average clustering coefficient of 0.59, and a modularity of 1.68 (Figure 7A). By calculating the between-module connectivity (Pi) and within-module connectivity (Zi) of the soil nitrogen-fixing bacterial network (Figure 7B), the results show that among the four types of network nodes, 96.97% of nodes were peripheral nodes (Zi ≤ 2.5, Pi ≤0.62), and there were no network hubs (Zi > 2.5, Pi >0.62) within the entire microbial network. However, the nitrogen-fixing bacterial network consisted of five between-module connectors (Zi ≤ 2.5, Pi >0.62), namely *Rhodospirillum centenum* of the Rhodospirillum genus, *Immundisolibacter cernigliae* of the Immundisolibacter genus, *Altererythrobacter* sp. *B11* of the Altererythrobacter genus, *Bradyrhizobium guangxiense* of the Bradyrhizobium genus, and one unidentified bacterium. There were two module hubs (Zi > 2.5, Pi ≤0.62), which included Bradyrhizobium oligotrophicum of the Bradyrhizobium genus and one unidentified bacterium. The study results of nitrogen response indicator species showed that the indicator species for nitrogen deposition was *Bradyrhizobium* sp. *BTAi1* from the Bradyrhizobium genus, with an Indval value of 0.995 and a *p* value of 0.022. The two indicator species for excessive nitrogen deposition were found to be *Bradyrhizobium japonicum* and *Bradyrhizobium* sp. *ORS 285* from the Bradyrhizobium genus.

## 4 Discussion

### 4.1 Effects of nitrogen deposition on the abundance of nitrogen-fixing bacterial communities

Previous studies showed that nitrogen-fixing bacterial communities were sensitive to nitrogen deposition, characterized by the fact that moderate nitrogen addition promotes the abundance of nitrogen-fixing bacteria, while high concentrations of nitrogen addition has an inhibitory effect on the abundance. Orr et al. (2012) found that moderate nitrogen addition could increase the content of organic carbon in soil as well as the abundance of nitrogen-fixing bacteria. Berthrong et al. (2014) showed that the nitrogen-fixing microorganisms were highly sensitive to soil nitrogen, and exhibited a decrease in the abundance of soil nitrogen-fixing microorganisms with an increase in the nitrogen deposition. Liu H. M. et al. (2019) conducted a simulated nitrogen deposition experiment in grasslands and found that the abundance of nitrogen-fixing microorganisms initially increased and later decreased with increasing nitrogen application. In this study, compared with the control, the low nitrogen treatment improved the diversity of the nitrogen-fixing bacterial community, while medium and high nitrogen treatments reduced their diversity, which is consistent with the above results. The reason for this outcome could be that low levels of nitrogen deposition alleviate the soil’s original nitrogen limitation, thus promoting carbon sequestration in the soil, increasing soil microbial carbon use efficiency and hence increasing the abundance of nitrogen-fixing bacteria (Wang et al., 2018; Li et al., 2021). As nitrogen deposition increases, the soil and atmospheric inorganic nitrogen reach a certain threshold, leading to reduced competitiveness of nitrogen-fixing bacterial communities, slowing nitrogen fixation rates. Furthermore, the increase in available nitrogen content in the soil may reduce the plants’ dependence on nitrogen-fixing microorganisms, inhibiting the activity of soil nitrogen-fixing microorganisms and leading to a decrease in the abundance of soil nitrogen-fixing microorganisms (Lu et al., 2022).

The nitrogen input threshold at which the abundance of nitrogen-fixing bacterial communities is suppressed is not yet unanimously concluded, and it is subject to differences in study sites, ecological types, and levels of disturbance. Liu M. et al. (2019) found that the abundance of nitrogen-fixing microorganisms appeared to be reduced when the nitrogen input was 300 kg N hm^−2^ a^−1^ in a study of nitrogen deposition in Baikal Needle Fescue Grassland, Inner Mongolia. Liu (2017) found that the abundance of nitrogen-fixing bacterial flora appeared to be reduced when the nitrogen input was 60 kg N hm^−2^ a^−1^ in a simulated nitrogen deposition in a fir plantation in Jiangxi province. Compton et al. (2004) found a suppressed threshold for nitrogen inputs in a simulated N deposition experiment in Harvard Forest and found that soil N-fixing bacteria and N-fixing gene expression was suppressed with N deposition above 50 kg N hm^−2^ a^−1^. Berthrong et al. (2014) found that soil N-fixing genes decreased in abundance and diversity with N additions up to 112 kg N hm^−2^ a^−1^ in *Pinus sylvestris* forest. In this study, when the N deposition of *C. oleifera* plantation reached 40 kg N hm^−2^ a^−1^, the diversity of soil nitrogen-fixing bacterial groups showed a decreasing trend and the community composition changed significantly. Compared with grassland and other forest types, *C. oleifera* plantations have the lowest N thresholds for soil N-fixing flora, which may be attributed to the fact that *C. oleifera* is an economic forest that requires long-term care by human beings, with a high degree of forest disturbance and frequent reorganisation of the soil flora resulting in a lack of stability in the structure of the microbial community itself. At the same time, in order to guarantee the yield of *C. oleifera* forests, the artificial care promotes the extremes in the distribution of resources (Deng et al., 2022), making *C. oleifera* forests less able to resist external environmental disturbances and more sensitive to N deposition.

### 4.2 Changes in community structure and assembly of nitrogen-fixing bacterial communities under nitrogen deposition

In this study, Bradyrhizobiaceae, a slow-growing rhizobium, was found to be the dominant bacterial family in the soil. Similar findings were confirmed by Zhao et al. (2020) in a study of nitrogen-fixing bacterial communities in 146 forest soils across the country, indicating that thermophilic slow-growing rhizobia were prevalent in forest soils and that community composition was more influenced by environmental factors than soil factors. By decomposing the β-diversity of nitrogen-fixing communities, we found that community differences caused by nitrogen deposition were determined by the species replacement process, which may be related to the environmental filtering and competition (Angeler, 2013; Legendre, 2014). With changes in the environmental conditions, the habitat in the rhizosphere soil correspondingly changes. When environmental parameters approach the optimal habitat for a particular genus, its growth and proliferation accelerate, an increase in the uptake of resources and living space is seen, and excludes the weaker communities, thereby leading to species replacement events. In this study, as the amount of nitrogen deposition increased, the proportion of Bradyrhizobiaceae gradually increased, while the proportion of Desulfobulbaceae decreased. It is speculated that these two microbial groups, as dominant nitrogen-fixing communities in the rhizosphere of *C. oleifera*, exhibit a complementary effect on ecological niches, which is more beneficial to the stability of nitrogen-fixing function (Wang et al., 2017).

The assembly process determines the distribution and abundance patterns of microbial communities. After community species succession, there is a complex interplay among the microbial communities, which requires the synergistic drive of plant–soil-microbe interactions. The formation of microbial community structure is a dynamic and complex process that requires the synergy of multiple soil variables, and nitrogen input plays a decisive role in the construction of nitrogen-fixing community structures (Coelho et al., 2009). Nitrogen input may affect the relative importance of the deterministic and stochastic processes, thus influencing the trajectory of community aggregation. Previous studies on the construction process of nitrogen-fixing microbial communities have shown that both deterministic and stochastic processes influence the community establishment, but the predominating process was disputed and closely related to the degree of environmental filtering and spatial heterogeneity (Liu S. et al., 2021; Liu W. et al., 2021). Some studies have suggested that excessive nitrogen deposition usually leads to an increase in ecosystem productivity, thereby increasing the importance of random assembly processes (Chase, 2010). Other researches indicate that nitrogen addition may increase the directed environmental filtering, facilitating better resource competition, which strengthens the importance of deterministic processes in forming community aggregation, thereby enhancing the convergence of community structure (Hautier et al., 2009; Conradi et al., 2017). In this study, nitrogen deposition treatments drove the community assembly, with the assembly predominantly governed by stochastic processes. This might be because the nitrogen content in *C. oleifera* soil was relatively low; although current high levels of nitrogen deposition have some environmental filtering effects on the rhizosphere microorganisms, they did not exhibit a strong selective pressure or significant microbial fitness differences. Therefore, the assembly process was still predominantly stochastic. We will continue to carry out nitrogen deposition studies in *C. oleifera* plantations, increase the nitrogen deposition levels continuously, and further explore the community assembly patterns and mechanisms driven by excessive nitrogen deposition for nitrogen-transforming microbial communities.

Nitrogen transformation is a complex process regulated by various microbes and enzyme activities, and soil enzymes are the metabolic driving force of soil organisms, with their activity being an important indicator reflecting the soil quality and fertility (Liu S. et al., 2021; Liu W. et al., 2021). In this study, urease was selected to measure the rate of conversion from organic nitrogen to inorganic nitrogen in ammonification, soil hydroxylamine reductase activity represents the loss of ammonia volatilization in soil nitrogen metabolism, and nitrate reductase and nitrite reductase activities measure the soil nitrogen retention capacity (Chen et al., 2002). The results show that the abundance of nitrogen-fixing bacterial communities was positively correlated with the activities of soil urease, nitrate reductase, nitrite reductase, and hydroxylamine reductase. It is speculated that low levels of nitrogen deposition increased the biomass and abundance of nitrogen-fixing bacterial communities in the soil and enhanced nitrogen-fixing capacity. *C. oleifera* is an ammonium-preferring plant, and low nitrogen input promotes productivity improvement and accelerates the absorption and transport of the ammonium nitrogen. When nitrogen deposition reaches a certain level, forest soil tends to a nitrogen-saturated state, resulting in carbon and phosphorus limitations and weakened soil respiration, which is not conducive to the growth and reproduction of nitrogen-fixing microorganisms and the synthesis of energy required for biological nitrogen fixation (Chen et al., 2020; Xing et al., 2022).

## 5 Conclusion

Nitrogen deposition impacts the nitrogen content in the soil, as well as the structure and diversity of nitrogen-fixing bacterial communities. In this study, we explored the impact of nitrogen deposition on the structure and function of nitrogen-fixing bacterial communities. We studied the response strategies of nitrogen-fixing bacterial communities to nitrogen feedback regulation in conditions with increased nitrogen content of both the air and soil. It was found that low nitrogen deposition was conducive to nitrogen fixation in mature *C. oleifera* plantations. Low nitrogen deposition treatments increased the diversity of soil nitrogen-fixing bacteria, while medium and high nitrogen treatments reduced their diversity. The differences in β-diversity of community composition were determined by the species replacement process. As the nitrogen deposition increased, the dominant soil nitrogen-fixing bacterial groups shifted from Desulfobulbaceae to Bradyrhizobiaceae. When nitrogen deposition was below 160 kg N·hm^−2^·a^−1^, the factors including the soil organic matter content, total nitrogen content, nitrate nitrogen content, ammonium nitrogen content, urease activity, soil pH value, and nitrate reductase activity influenced the construction of the nitrogen-fixing bacterial community, but stochastic processes remained the predominant factor. Further, it was found that the strains of *Bradyrhizobium japonicum* and *Bradyrhizobium* sp. *ORS 285* can serve as indicator species for excessive nitrogen deposition, providing technical support for precise management of soil nitrogen under changing nitrogen deposition conditions.

## Data availability statement

The raw data supporting the conclusions of this article will be made available by the authors, without undue reservation.

## Author contributions

CL: Data curation, Investigation, Writing – original draft, Writing – review & editing. ZH: Data curation, Investigation, Writing – original draft, Writing – review & editing. YC: Data curation, Supervision, Writing – review & editing. YX: Investigation, Writing – original draft. WT: Investigation, Writing – original draft. LC: Funding acquisition, Project administration, Writing – review & editing.
